# Assessment of the relationship between organ donation attitudes and religious beliefs among postgraduate students

**DOI:** 10.3389/fpubh.2025.1596640

**Published:** 2025-07-17

**Authors:** Umit Apak, Sami Akbulut, Zeynep Kucukakcali, Hasan Saritas

**Affiliations:** ^1^Transplantation Coordinator Program, Inonu University Liver Transplant Institute, Malatya, Türkiye; ^2^Department of Surgery and Liver Transplant Institute, Inonu University Faculty of Medicine, Malatya, Türkiye; ^3^Department of Biostatistics and Medical Informatics, Inonu University Faculty of Medicine, Malatya, Türkiye; ^4^Department of Public Health, Inonu University Faculty of Medicine, Malatya, Türkiye; ^5^Department of Surgical Nursing, Siirt University Faculty of Health Science, Siirt, Türkiye

**Keywords:** attitudes, knowledge, organ donation, organ donation attitude scale, postgraduate students, religious beliefs

## Abstract

**Background:**

Organ donation is a critical public health issue, and understanding the factors influencing individuals' knowledge, attitudes, and awareness is essential. To address this, we conducted a descriptive and analytical study among postgraduate students, aiming to evaluate the relationship between their knowledge, attitudes, and awareness of organ donation and their religious beliefs.

**Methods:**

A survey-based cross-sectional study was conducted among about 500 postgraduate students at Inonu University Health Sciences Institute. A demographic information form, an organ donation knowledge form, and the validated Turkish version of the Organ Donation Attitude Scale (ODAS) were used. Data were collected online via Google Forms, except for 10 students who filled out paper forms due to email issues. Independent variables included age, marital status, education programs, alcohol and cigarette use, and awareness of organ donation, while dependent variables were ODAS total and subdimension scores.

**Results:**

A total of 324 postgraduate students completed the survey. Despite 96.5% recognizing the necessity of organ donation, only 16.9% reported having registered as donors. Religious beliefs were important for 92.5% of postgraduate students, influenced major decisions for 62.2%, and 65.8% believed organ donation was compatible with Islam. The ODAS total scores showed no significant differences based on gender (*p* = 0.073), marital status (*p* = 0.483), education program (*p* = 0.051), or the influence of religious beliefs on life decisions (*p* = 0.135). Doctoral postgraduate students were more aware of the fatwa on organ donation (*p* = 0.010). Postgraduate students who had not donated an organ were significantly more likely to believe that brain death is reversible (*p* < 0.001), to disapprove of organ donation from a Muslim to a non-Muslim (*p* = 0.004), and to consider organ donation incompatible with Islam (*p* < 0.001). The Cronbach's alpha value of the ODAS scale was 0.841, indicating good internal consistency.

**Conclusion:**

Although religious beliefs influenced major life decisions for most postgraduate students, they did not significantly alter attitudes toward organ donation, as measured by ODAS scores. Misconceptions about brain death and religious permissibility persist, highlighting the need for targeted educational programs, especially considering that postgraduate students, as future health professionals, can play a crucial role in promoting organ donation awareness.

## 1 Introduction

Organ donation refers to the act of donating the tissues and organs of individuals who have been medically proven brain-dead to others, either with their own consent or with the consent of their close relatives. Therefore, organ donation is an important public health issue that allows people with chronic diseases to hold on to life when viewed from a societal perspective. Organ donation is crucial for modern healthcare systems, yet religious beliefs often shape individuals' willingness to donate (1, 2). Despite all the organ donation campaigns that claim organ donation is important for humanitarian, moral, and conscientious reasons, the current number of organ donations remains far below the number of organs requested—that is, the number of patients on the waiting list (3). Therefore, it is crucial to develop effective strategies to promote organ donation by analyzing the cultural structure and beliefs of society (2). Organ donation and cadaveric organ transplantation are relatively high in western countries. However, in underdeveloped and developing countries, organ donation is significantly insufficient, resulting in a reliance on living organ donors to meet the organ need. Scientific studies have revealed many factors affecting organ donation, with religious beliefs, cultural values, and differences in educational systems being the most emphasized (4).

Ethical, cultural, and religious factors deeply intertwine with the decision to donate organs, significantly influencing individuals' attitudes and willingness to donate. Inadequate donation rates have led to extensive research on the factors that influence organ donation, including ethical considerations, public perceptions, legal frameworks, and particularly religious beliefs. Understanding these elements is essential to developing effective strategies to increase organ donation rates worldwide.

One of the main ethical issues affecting organ donation is the balance between altruism and the perceived commodification of human organs. People often cite altruism as the primary motivation for organ donation, and research indicates that individuals who perceive organ donation as a selfless act are more inclined to consent to organ donation (5, 6). However, public perceptions of organ donation can significantly influence willingness to donate. For instance, studies have shown that public awareness campaigns addressing misconceptions about organ donation can enhance the intention to donate organs (7). Such initiatives are essential for altering public perceptions and increasing total donation rates.

Legal frameworks also play an important role in shaping organ donation rates. Different countries use a variety of consent models, such as opt-in and opt-out systems. Opt-out systems, which assume individuals' consent unless they explicitly refuse, tend to yield higher donation rates compared to opt-in systems, as demonstrated by research (8, 9). For example, the introduction of an opt-out system in Wales has led to increased consent rates for organ donation (10).

Family dynamics and the healthcare professionals' role are also critical factors in the organ donation process. Studies have shown that the timing and manner in which healthcare providers approach families about organ donation can affect consent rates. In particular, the involvement of a specialist nurse and coordinator in the interview can lead to higher consent rates (11). In addition, family members' beliefs and attitudes toward organ donation can facilitate or hinder the decision to donate (12). Education for healthcare professionals can increase the likelihood of obtaining consent from families.

In predominantly Muslim countries such as Türkiye, where religious interpretations can strongly influence health decisions, investigating the compatibility of organ donation with religious beliefs is particularly important (4, 13–15). Numerous studies have demonstrated that religious beliefs play a pivotal role in shaping individuals' attitudes toward organ donation, with cultural and theological interpretations significantly influencing willingness to donate. For instance, recent research has shown that while the three major monotheistic religions (Christianity, Islam, and Judaism) generally support organ donation, practical willingness remains low due to varying interpretations and cultural practices (16–19). In some societies, in addition to the factors mentioned above, cultural and religious beliefs also significantly affect attitudes toward organ donation (14, 20). Religion often plays a central role in the maturation of individuals' moral values and ethical perspectives, which can affect their willingness to donate organs (21). Rasheed and Padela (22) reported in their study that religious beliefs significantly affect individuals' attitudes toward organ donation, with some religious groups expressing more positive attitudes than others. Similarly, Dibaba et al. (23) emphasized the importance of religious leaders and institutions in shaping organ donation attitudes in communities. In some cultures, there is a strong emphasis on the sanctity of the body after death, which may conflict with the acceptance of organ donation (12).

Increasing organ donation requires a comprehensive strategy that takes into account ethical motivations, public perceptions, legal frameworks, family dynamics, and cultural beliefs. By addressing these interconnected factors through comprehensive education and policy initiatives, it is possible to increase the desire to donate organs and ultimately save more lives. Postgraduate students, particularly those in health-related fields, are pivotal as future health educators and policy influencers; understanding their perspectives can help shape targeted interventions to boost organ donation rates. This study aims to explore the attitudes of postgraduate students—characterized by high educational attainment and diverse religious beliefs—toward organ donation and examine the relationship between these attitudes and religious beliefs. This study can provide valuable information for developing targeted intervention strategies and educational campaigns, particularly within healthcare education contexts, to increase organ donation rates among postgraduate students. In addition, the study aims to increase awareness and initiate a social dialogue by emphasizing the complex relationship between organ donation attitudes and religious beliefs in light of the information provided.

## 2 Materials and methods

### 2.1 Type of research

We conducted this survey-based cross-sectional research to assess the relationship between postgraduate students' attitudes toward organ donation and their religious beliefs.

### 2.2 Place and time of the research

This survey-based cross-sectional research was conducted online on students receiving postgraduate education at Inönü University Institute of Health Sciences between March 2022 and September 2023. The study's population consisted of approximately 500 postgraduate students who were pursuing master's and doctoral degrees in 35 departments of the Institute, primarily affiliated with the Faculty of Physical Education and Sports, Faculty of Medicine, Faculty of Nursing, Faculty of Health Sciences, and Faculty of Pharmacy.

### 2.3 Study population and sample size

The study aimed to include all postgraduate students who met the inclusion criteria, without performing a priori sample size calculation. Since the study aimed to include the entire population of postgraduate students, no prior power analysis was conducted. However, *post-hoc* power analysis revealed a statistical power of ~100% for both correlation analyses and three-group comparisons, confirming that the sample size was adequate to detect meaningful differences.

### 2.4 Inclusion and exclusion criteria

The inclusion criteria were clearly defined as postgraduate students who were actively enrolled during the study period, voluntarily participated, and completed all survey items. The exclusion criteria included foreign students lacking sufficient proficiency in the Turkish language and those who did not complete the survey. Google Forms were sent via email to all postgraduate students who met the inclusion criteria. However, 10 postgraduate students who experienced email access issues completed a traditional paper-based version of the survey. Responses from postgraduate students who completed the survey were included in the study, resulting in 324 valid responses out of around 500 invited postgraduate students.

### 2.5 Conceptual definitions of key terms

Four fundamental key constructs were identified to evaluate the factors influencing organ donation attitudes comprehensively. “Knowledge” refers to the level of information and understanding about organ donation. “Awareness” indicates the recognition and acknowledgment of the importance of organ donation and related processes. “Attitudes” represent the positive or negative perceptions toward organ donation. Finally, “Behaviors” encompass the actual actions or intentions to donate organs.

### 2.6 Data collection

Data were collected online from March 2022 to September 2023 using a structured survey form designed on the Google platform. The survey consisted of three main components: the demographic information form, the organ donation information form, and the Organ Donation Attitude Scale (ODAS), all developed based on a comprehensive literature review. The survey was self-administered, and all items were mandatory to ensure data completeness. To ensure clarity and validity, the survey form was pilot tested with a small group of postgraduate students, allowing for necessary adjustments before the full implementation. Since data collection was conducted online, there is a potential selection bias toward students who are more digitally engaged or interested in health education topics.

### 2.7 Demographic information form

This section consists of 12 questions regarding the sociodemographic characteristics of the postgraduate students who consented to participate in the study, including gender, age, marital status, faculty, education programs, and lifestyle habits such as smoking and alcohol consumption.

### 2.8 Information form regarding organ donation

This section comprises 12 questions intended to assess postgraduate students' knowledge, attitudes, and behaviors about organ donation and transplantation, as well as the effect of Islamic beliefs on organ donation.

### 2.9 Organ donation attitude scale (ODAS)

Parisi and Katz (24) developed the ODAS scale in 1986, and Kent and Owens (25) revised it in 1995. The scale contains a total of 46 items, 23 positive and 23 negative, indicating attitudes toward organ donation. The Turkish validity and reliability study was conducted by Sayin (26) in 2015. Yazici Sayin prepared the Turkish form of the scale in a six-point Likert type (strongly agree: 6 points, slightly agree: 5 points, very little agree: 4 points, partially disagree: 3 points, mostly disagree: 2 points, strongly disagree: 1 point), with a total of 40 questions. Sayin (26) divided the scale into three subdimensions, one positive and two negative, based on the content of the questions. The first subdimension, consisting of positive statements about organ donation, consists of 20 items indicating people's helpfulness and moral values/beliefs regarding organ donation (Humanity and moral conviction: HMC; Table 1). The second subdimension consists of a total of 10 questions highlighting the fear of medical neglect (Fears of medical neglect: FMN; Table 2). The third subdimension consists of a total of 10 questions highlighting the fear of bodily mutilation (Fears of bodily mutilation: FBM; Table 3). The scale's positive attitudes yield scores ranging from 20 to 120. Similarly, the total negative attitude score is between 20 and 120. High positive and low negative scores indicate that voluntary attitudes toward organ donation are strong (26).

**Table 1:** Demographic characteristics of postgraduate students.

**Demographic features**	**Results**
Age (yr) [median (min–max)]	29 (21–58)
Gender [number (%)]	
Male	126 (38.9)
Female	198 (61.1)
Marital status [number (%)]	
Single	174 (54)
Married	148 (46)
Postgraduate education program? [number (%)]	
Master's degree	135 (42.6)
Doctorate degree	182 (57.4)
Smoking [number (%)]	
Yes	53 (16.5)
No	246 (76.6)
Exsmoker	22 (6.9)
Alcohol use ? [number (%)]	
Yes	31 (9.6)
No	285 (88.5)
Exdrinker	6 (1.9)

**Table 2:** Views of postgraduate students on organ donation.

**Variables**	***n* (%)**
Do you think organ donation is necessary?	
No	11 (3.5)
Yes	307 (96.5)
Have you donated your organs?	
Yes	54 (16.9)
Undecided	225 (70.3)
I don't think so	41 (12.8)
In your opinion, what are the factors that affect organ donation? (one or more options can be selected)	
My organs are a hope for life for other people	101 (69.7)
My family and friends have also donated organs	2 (1,4)
My religious beliefs and the fatwas of the Presidency of Religious Affairs	8 (5.5)
I or someone in my family may need an organ transplant in the future	34 (23.4)
What are your reasons to refuse organ donation? (one or more options can be selected)	
I have many fears	88 (27.4)
I have no specific reason	103 (32.1)
I have many health problems	22 (6.9)
Religious beliefs	32 (10.0)
I don't know where to apply for donations	12 (3.7)
I have concerns about the accuracy of the diagnosis of brain death	37 (11.5)
Body integrity can deteriorate after death	19 (5.9)
I have a serious chronic disease (one or more)	8 (2.3)

**Table 3:** Knowledge and attitudes of postgraduate students on the subject of “the effects of religious beliefs on organ donation.”

**Variables**	***n* (%)**
Do religious beliefs have a place in your life?	
No	24 (7.5)
Yes	296 (92.5)
Do your religious beliefs influence your important decisions?	
No	29 (9.1)
Partially	92 (28.7)
Yes	199 (62.2)
Do you think organ donation is appropriate for Islamic belief?	
No	23 (7.2)
No idea	86 (27.0)
Yes	210 (65.8)
Do you think the Presidency of Religious Affairs has a fatwa on organ donation?	
No	7 (2.2)
No idea	195 (60.9)
Yes	118 (36.9)
Do you think it is appropriate for a non-Muslim's organs to be used for a Muslim?	
No	9 (2.8)
No idea	56 (17.4)
Yes	256 (79.8)
Do you think it is appropriate for a Muslim's organs to be used for a non-Muslim?	
No	10 (3.1)
No idea	55 (17.2)
Yes	255 (79.7)
Do you think brain death can be reversible?	
No	184 (57.5)
No idea	70 (21.9)
Yes	66 (20.6)

### 2.10 Research variables

The independent variables include age, gender, marital status, smoking, education programs, and postgraduate students' opinions on organ donation, while the dependent variables include the total and subdimensions of the ODAs scale. It should be noted that some participants did not respond to certain questions; therefore, the total number of respondents varies across items and may be lower than the overall sample size of 324.

### 2.11 Statistical analysis

IBM SPSS Statistics for Windows, Version 26.0 (IBM Corporation, Armonk, NY, USA) was used to analyze the data. Categorical data were summarized using numbers (percentages), while quantitative data were presented as medians (minimum-maximum). The Kolmogorov-Smirnov test was applied to assess whether the data conformed to a normal distribution. The Mann-Whitney U test, Kruskal-Wallis test, Pearson chi-square test, Yates corrected chi-square test, and Fisher's exact chi-square test were utilized as appropriate. To evaluate the relationship between the dependent variable and independent variables, a linear regression model was employed. This model allowed for estimating the extent to which each predictor variable independently contributed to the variation in ODAS scores while controlling for potential confounders. By applying the linear regression model, significant predictors that influence organ donation attitudes among postgraduate students were identified. The reliability of the postgraduate students' responses to the questions comprising the total and three subdimensions of the applied scale was analyzed using Cronbach's alpha coefficient. Furthermore, the correlation coefficient was employed to determine whether there was a correlation between the subdimensions of the scale and the total scores. In the statistical analyses performed, *p* < 0.05 was considered statistically significant.

### 2.12 Ethical considerations

Institutional permission for the study was first granted by the Inonu University Health Sciences Institute (Approval no. 144138, dated 11 February 2022). Subsequently, the study was reviewed and approved by the Inonu University Non-Interventional Clinical Research Ethics Committee (Approval no. 3153, dated 08 March 2024). After identifying the postgraduate student participants, they were provided with online information detailing the study's goals and objectives. Participants were informed of their right to withdraw from the study at any time, and written informed consent was obtained to confirm their voluntary participation.

## 3 Results

As shown in Table 1, the socio-demographic characteristics of the postgraduate students. The study determined that the postgraduate students' median age was 29 years (min-max: 21–58 years), with 61.1% being female, 54% being single, 57.4% enrolled in a doctoral program, 76.6% not smoking, and 88.5% not consuming alcohol.

Table 2 demonstrates the postgraduate students' knowledge and opinions on organ donation. Despite 96.5% recognizing the necessity of organ donation, only 16.9% reported having donated organs. Additionally, 70.3% stated that they are still undecided about organ donation, while 12.8% stated that they have never considered it. Most postgraduate students (69.7%) thought it was appropriate for others to benefit from their organs after death, while 23.4% supported organ donation because they or their family might need transplants. Among those hesitant to donate, 32.1% did not have a specific reason, 27.4% stated fear as the reason, and 10.0% cited religious beliefs.

As shown in Table 3, the postgraduate students' knowledge, attitudes, and behaviors concerning the influence of religious beliefs on organ donation indicate that 92.5% of postgraduate students reported that their religious beliefs play a significant role in their lives. Additionally, 62.2% stated that religious beliefs influence their important decisions, while 65.8% believed that organ donation complies with Islamic belief. Notably, 60.9% expressed that they have no idea whether the Presidency of Religious Affairs has a fatwa on organ donation. Furthermore, 79.8% considered it appropriate for the organs of a non-Muslim to be used for a Muslim when necessary, and 79.7% found it acceptable for the organs of a Muslim to be used for a non-Muslim when necessary. Lastly, 57.5% acknowledged that there is no way to bring an individual who is brain-dead back to life.

As shown in Table 4, the postgraduate students' responses to the 20-item HMC subscale of the ODAS scale indicate a strong positive attitude toward organ donation. The three most common responses reflect a sense of altruism and moral conviction. Specifically, 41.4% of postgraduate students strongly agreed with the statement, “A person willing to donate is almost a hero.” Similarly, 41.4% strongly agreed with the statement, “Donating a body part would enable that part of myself to remain alive after my death.” Furthermore, 66.7% answered “strongly agree” to the statement, “By agreeing to donate organs at death, one sets a good example for others to follow.” A considerable proportion of postgraduate students (66.1%) strongly agreed with the statement, “Deciding to donate one's organs at death adds extra meaning to life.” Additionally, 39.3% strongly agreed that “Organ donation endows death with more meaning and worth.” The perception of organ donation as a moral duty was reflected by 43.7% of postgraduate students strongly agreeing with the statement, “Vowing to donate organs at death is a highly moral act.” The idea of social respect linked to organ donation was moderately supported, with 29.9% slightly agreeing that “Vowing to donate organs at death makes one more respected and admired by family and friends.” Religious motivations also played a role, as 22.0% strongly agreed that “Donating organs is a way to thank God.” Positive perceptions about the impact of organ donation were highlighted by 57.1% of postgraduate students strongly agreeing with the statement, “Hearing about people whose lives were saved after receiving an organ makes me think about the importance of donating my organs after death.” Additionally, 52.5% strongly agreed that “Donating organs after death is a way to ensure that some parts of the body are useful,” and 56.8% strongly agreed that “A person who allows a part of their body to be transplanted to someone else is truly giving a precious gift. A significant number of postgraduate students (61.3%) strongly agreed that “By deciding to donate my organs after death, I would be providing hope for survival to some people.” The view that organ donors are unique individuals was supported by 48.1% strongly agreeing with the statement, “Organ donors are special people.” Furthermore, 64.9% strongly agreed that “Organ donation benefits all humanity,” and 61.0% strongly agreed that “Life is too precious to end due to an unhealthy heart or kidney, especially if organ donation can solve the problem.” The sense of contributing to others' survival was emphasized by 56.4% strongly agreeing with the statement, “I can ensure that someone else lives by informing them that my organs can be taken after my death.” Additionally, 45.2% strongly agreed that “Donating an organ after my death would make me proud of myself.”

**Table 4:** Distributions of responses given by postgraduate students to the HMC subdimension of the ODAS scale (the three most preferred options).

**Items**	**Response options**	***n* (%)**
HMC- A person willing to donate is almost a hero	Strongly agree	132 (41.4)
	Slightly agree	102 (32.0)
	Very little agree	27 (8.5)
HMC- Donating a body part would enable that part of myself to remain alive after my death	Strongly agree	132 (41.4)
	Slightly agree	83 (26.0)
	Strongly disagree	40 (12.5)
HMC- By agreeing to donate organs at death, one sets a good example for others to follow	Strongly agree	212 (66.7)
	Slightly agree	76 (23.9)
	Very little agree	11 (3.5)
HMC- Deciding to donate one's organs at death adds extra meaning to life	Strongly agree	211 (66.1)
	Slightly agree	69 (21.6)
	Very little agree	14 (4.4)
HMC- Organ donation endows death with more meaning and worth	Strongly agree	125 (39.3)
	Slightly agree	81 (25.5)
	Very little agree	44 (13.8)
HMC- Vowing to donate organs at death is a highly moral act	Strongly agree	139 (43.7)
	Slightly agree	91 (28.6)
	Very little agree	35 (11.0)
HMC- Vowing to donate organs at death makes one more respected and admired by family and friends	Strongly agree	79 (24.8)
	Slightly agree	95 (29.9)
	Very little agree	67 (21.1)
HMC- Organ donation is a way of being grateful for God	Strongly agree	69 (22.0)
	Slightly agree	67 (21.3)
	Strongly disagree	67 (21.3)
HMC- Hearing about people whose lives were saved after the receipt of an organ makes me think about the importance of donating my organs after death	Strongly agree	182 (57.1)
	Slightly agree	72 (22.6)
	Very little agree	32 (10.0)
HMC- Donating organs at death is a way of putting some parts of the body to beneficial use	Strongly agree	166 (52.5)
	Slightly agree	82 (25.95)
	Very little agree	29 (9.2)
HMC- The person who offers a part of his or her body for transplantation is making a really precious gift	Strongly agree	179 (56.8)
	Slightly agree	76 (24.1)
	Very little agree	31 (9.8)
HMC- People have a moral responsibility to donate some of their body parts to people in need	Strongly agree	145 (46.0)
	Slightly agree	103 (32.7)
	Very little agree	35 (11.1)
HMC- By agreeing to donate my organs after death, I am giving some people hope for survival	Strongly agree	192 (61.3)
	Slightly agree	73 (23.3)
	Very little agree	25 (8.0)
HMC- Organ donors are special people	Strongly agree	149 (48.1)
	Slightly agree	86 (27.7)
	Very little agree	38 (12.3)
HMC- Organ donation benefits the whole of humanity	Strongly agree	203 (64.9)
	Slightly agree	66 (21.1)
	Very little agree	23 (7.35)
HMC- Life is much too valuable to be cut short by a bad heart or kidneys, especially when organ donation can help to solve the problem	Strongly agree	189 (61.0)
	Slightly agree	71 (22.9)
	Very little agree	22 (7.1)
HMC- By donating a body part after my death, I could keep another person living	Strongly agree	176 (56.4)
	Slightly agree	76 (24.4)
	Very little agree	25 (8.0)
HMC- By donating an organ at death, one can offer someone a better chance of being cured	Strongly agree	211 (67.6)
	Slightly agree	71 (22.8)
	Very little agree	15 (4.8)
HMC- Donating an organ after my death would make me feel proud of myself	Strongly agree	142 (45.2)
	Slightly agree	84 (26.8)
	Very little agree	42 (13.4)
HMC- Promising to donate is a genuine and unselfish act	Strongly agree	197 (62.9)
	Slightly agree	71 (22.7)
	Very little agree	24 (7.7)

HMC, Humanity and moral convinction.

Finally, 62.9% of postgraduate students strongly agreed with the statement, “It is a sincere and selfless act to declare that you will donate an organ,” while 67.6% strongly agreed that “You can increase someone's chance of recovery by donating an organ at the time of death.”

As demonstrated in Table 5, the postgraduate students' responses to the 10-item FMN subdimension of the ODAS scale reveal a generally low level of fear related to medical neglect. The three most common responses indicate a strong disagreement with concerns about inadequate medical care or unethical practices. Specifically, 44.3% of postgraduate students strongly disagreed with the statement, “Organ donors cannot control which organs will be taken even when specified in advance.” Additionally, 33.5% strongly disagreed with the statement, “Medical doctors who remove organs do not treat the body in a dignified manner.”

**Table 5:** Distributions of responses given by postgraduate students to the FMN subdimension of the ODAS scale (the three most preferred options).

**Items**	**Response options**	***n* (%)**
FMN- Organ donors cannot control which organs will be taken even when specified in advance	Partially disagree	48 (15.9)
	Mostly disagree	38 (11.95)
	Strongly disagree	141 (44.3)
FMN- Medical doctors who remove organs do not treat the body in a dignified manner	Partially disagree	61 (19.3)
	Mostly disagree	51 (16.1)
	Strongly disagree	106 (33.5)
FMN- Whole bag of tricks of medical will not be used to save the life of someone who has signed a donor card	Partially disagree	37 (11.6)
	Mostly disagree	44 (13.8)
	Strongly disagree	171 (53.8)
FMN- A person will be less likely to receive adequate medical care after signing a donor card	Partially disagree	26 (8.2)
	Mostly disagree	58 (18.4)
	Strongly disagree	175 (55.4)
FMN- There is a good chance that doctors will be more likely to prematurely declare the death of a person who has signed a donor card	Very little agree	35 (11.0)
	Mostly disagree	52 (16.4)
	Strongly disagree	162 (51.1)
FMN- Organ donation should not be considered because the body is a God entrust and has religious meaning after death	Partially disagree	35 (11.1)
	Mostly disagree	63 (19.9)
	Strongly disagree	158 (50.0)
FMN- A potential donor's death will be met by pleasure rather than by vigorous medical treatment by doctors	Slightly agree	42 (13.4)
	Mostly disagree	46 (14.6)
	Strongly disagree	128 (40.8)
FMN- A person who intends to donate their body parts at death increases the likelihood that one will be pronounced dead even though one is still alive	Very little agree	26 (8.4)
	Mostly disagree	57 (18.3)
	Strongly disagree	161 (51.8)
FMN- By signing a donor card, doctors might do something to me before I am really dead	Slightly agree	25 (8.0)
	Mostly disagree	53 (17.0)
	Strongly disagree	172 (55.3)
FMN- Even if special precautions were taken to protect the life of a person who has signed a donor card, there is still a chance that their life will be taken to save the life of a rich or important person	Very little agree	45 (14.4)
	Mostly disagree	51 (16.4)
	Strongly disagree	108 (34.6)

FMN, Fears of medical neglect.

Moreover, 53.8% of the postgraduate students strongly disagreed with the statement, “A person who has signed a donor card will not receive adequate medical care,” highlighting a low level of fear regarding medical neglect after signing a donor card. Similarly, 55.4% of postgraduate students strongly disagreed with the statement, “A person will be less likely to receive adequate medical care after signing a donor card.”

In terms of perceptions about premature death declaration, 51.1% of postgraduate students strongly disagreed with the statement, “There is a good chance that doctors will be more likely to prematurely declare the death of a person who has signed a donor card.” Additionally, 50.0% of postgraduate students strongly disagreed with the statement, “Organ donation should not be considered because the body is a God-entrust and has religious meaning after death.”

Regarding potential biases in medical treatment, 40.8% of postgraduate students strongly disagreed with the statement, “A potential donor's death will be met by pleasure rather than by vigorous medical treatment by doctors.” Furthermore, 51.8% of postgraduate students strongly disagreed with the statement, “A person who intends to donate their body parts at death increases the likelihood that one will be pronounced dead even though one is still alive.” The concern that medical professionals might act prematurely was also addressed, with 55.3% of postgraduate students strongly disagreeing with the statement, “By signing a donor card, doctors might do something to me before I am really dead.” Lastly, 34.6% of postgraduate students strongly disagreed with the statement, “Even if special precautions are taken to protect the life of a person who has signed a donor card, there is still a chance that their life will be taken to save the life of a rich or important person.”

As presented in Table 6, the responses to the 10-item FBM subdimension of the ODAS scale indicate generally low levels of fear related to bodily mutilation or loss of body integrity. The three most common responses reflect a strong disagreement with concerns about bodily disfigurement or loss of self-identity. Specifically, 37.6% of postgraduate students strongly disagreed with the statement, “Organ donation leaves the body disfigured.” Additionally, 62.3% strongly disagreed with the statement that “An intact body is necessary for life after death.”

**Table 6:** Distributions of responses given by postgraduate students to the FBM subdimension of the ODAS scale (the three most preferred options).

**Items**	**Response options**	***n* (%)**
FBM- Organ donation leaves the body disfigured	Slightly agree	55 (17.2)
	Mostly disagree	51 (16.0)
	Strongly disagree	120 (37.6)
FBM- An intact body is needed for the life after death	Partially disagree	29 (9.1)
	Mostly disagree	48 (15.1)
	Strongly disagree	198 (62.3)
FBM- Other members of my family would object to me signing an organ donor card	Slightly agree	70 (22.0)
	Very little agree	49 (15.4)
	Strongly disagree	82 (25.8)
FBM- Preparing to become an organ donor brings to mind unpleasant thoughts of my own death	Slightly agree	64 (20.2)
	Very little agree	50 (15.8)
	Strongly disagree	89 (28.1)
FBM- The surest way to bring about my own death is to make plans for it, like signing a donor card	Strongly agree	32 (10.22)
	Mostly disagree	49 (15.7)
	Strongly disagree	145 (46.3)
FBM- Promising to donate my organs upon my death makes me feel uncomfortable	Very little agree	57 (18.3)
	Mostly disagree	56 (18.0)
	Strongly disagree	96 (30.9)
FBM- When I die I want the whole of my body to die with me	Partially disagree	38 (12.3)
	Mostly disagree	63 (20.3)
	Strongly disagree	120 (38.7)
FBM- A person with someone else's heart, eyes, kidney etc. is not the same person	Partially disagree	20 (6.4)
	Mostly disagree	60 (19.2)
	Strongly disagree	186 (59.6)
FBM- The thought of my body being cut up or taken apart after I'm gone makes me feel uneasy	Slightly agree	68 (21.8)
	Very little agree	47 (15.1)
	Strongly disagree	101 (32.4)
FBM- When I die I want to be buried whole and with all my original parts	Partially disagree	45 (14.5)
	Mostly disagree	54 (17.4)
	Strongly disagree	96 (30.9)

FBM, Fears of bodily mutilation.

Regarding social and familial perceptions, 22.0% slightly agreed with the statement, “Other members of my family would object to me signing an organ donor card.” However, 28.1% strongly disagreed with the statement, “Preparing to become an organ donor brings to mind unpleasant thoughts of my own death.”

Based on the postgraduate students' responses, a significant proportion, 46.3% strongly disagreed with the statement, “The surest way to bring about my own death is to make plans for it, like signing a donor card.” Similarly, 30.9% strongly disagreed with the statement, “Promising to donate my organs upon my death makes me feel uncomfortable.”

The perception of maintaining the body's wholeness after death was addressed, with 38.7% strongly disagreeing with the statement, “When I die, I want the whole of my body to die with me.” Furthermore, 59.6% strongly disagreed with the statement, “A person with someone else's heart, eyes, kidney, etc. is not the same person.”

The idea of post-mortem body disassembly causing discomfort was also examined. 32.4% strongly disagreed with the statement, “The thought of my body being cut up or taken apart after I'm gone makes me feel uneasy.” Additionally, 30.9% strongly disagreed with the statement, “When I die, I want to be buried whole and with all my original parts.”

Table 7 illustrates the statistical relationship between the four independent variables of postgraduate students and the ODAS total and subdimension scores. No significant difference was observed between female and male genders in terms of total (*p* = 0.073), HMC (*p* = 0.609), FMN (*p* = 0.936), and FBM (*p* = 0.799) scores. Similarly, there was no significant difference between married and single postgraduate students for total (*p* = 0.483), HMC (*p* = 0.376), FMN (*p* = 0.602), and FBM (p =0.631) scores. When comparing master's and doctorate students, the analysis revealed no significant difference in total (*p* = 0.051), FMN (*p* = 0.310), and FBM (*p* = 0.679) scores, while the HMC score of doctorate students was significantly higher (*p* = 0.016). Regarding religious beliefs, no significant difference was found between postgraduate students with and without religious beliefs in terms of total (*p* = 0.0135), HMC (*p* = 0.331), FMN (*p* = 0.923), and FBM (p =0.577) scores. A linear regression analysis was performed using the independent variables listed in Table 7. The results indicated that only the gender variable emerged as an independent factor influencing the total ODAS score (*p* = 0.045; Durbin-Watson value: 1.863).

**Table 7:** Comparison of some independent variables according to the total and subdimension scores of the ODAS scale.

**Variables [median (IQR)]**		**Total score**		**HMC score**		**FMN score**		**FBM score**	
		**Score**	***p***	**Score**	***p***	**Score**	***p***	**Score**	***p***
Gender	Male	146 (25)	0.073	102 (26)	0.609	20 (14)	0.936	25 (18)	0.799
	Female	151 (29)		105 (22)		22 (16)		27 (15)	
Marital status	Single	150 (27)	0.483	104 (22)	0.376	22 (14)	0.602	26 (16)	0.631
	Married	149 (27)		145 (26)		20 (19)		26 (17)	
Postgraduate education program	Master's degree	151 (30)	0.051	103 (22)	0.016	24 (14)	0.310	27 (15)	0.679
	Doctorate degree	149 (26)		105 (26)		19 (16)		26 (16)	
Do religious beliefs have a place in your life?	No	149 (19)	0.135	97 (32)	0.331	21 (12)	0.923	27 (19)	0.577
	Yes	150 (28)		105 (23)		20(15)		26 (16)	

Mann Whitney *U* test, HMC, Humanity and moral convinction; FMN, Fears of medical neglect; FBM, Fears of bodily mutilation.

Table 8 highlights the association between organ donation status and ODAS scores. Postgraduate students were grouped according to their response to the question, “Have you donated an organ?” and their total score, HMC score, FMN score, and FBM score were compared. A statistically significant difference (*p* = 0.042) was identified between the HMC score and organ donation status, while the remaining scores showed no significant difference.

**Table 8:** Comparison of total and subdimension scores of ODAS scale according to the answers given to the question “Have you donated an organ?.”

**Variables [median (IQR)]**	**Yes**	**No, I don't think so**	**No, undecided**	***p***
Total Score	149 (22)	148 (28)	150 (28)	0.840
HMC Score	97 (25)	104 (21)	105 (24)	0.042
FMN Score	22 (14)	18 (17)	20 (16)	0.463
FBM Score	29 (13)	23 (25)	25 (16)	0.052

Table 9 presents the distribution of responses to seven questions aimed at assessing postgraduate students' knowledge and attitudes toward organ donation by gender. A significant difference was found only in response to the question, “Do you think organ donation is appropriate for Islamic belief?” (*p* = 0.014). Notably, 75.2% of men answered “yes,” whereas 32.5% of women indicated “no idea.”

**Table 9:** Comparison of knowledge and attitudes of postgraduate students regarding organ donation according to gender.

**Items**	**Response**	**Male [n (%)]**	**Female [n (%)]**	***p***
Do religious beliefs have a place in your life?	No	13 (10.4)	11 (5,6)	0.174
	Yes	112 (89.6)	184 (94.4)	
Do your religious beliefs influence your important decisions?	No	15 (11.9)	14 (7.2)	0.351
	Partially	36 (28.6)	56 (28.9)	
	Yes	75 (59.5)	124 (63.9)	
Do you think organ donation is appropriate for Islamic belief?	No	8 (6.4)	15 (7.7)	0.014
	No idea	23 (18.4)	63 (32.5)	
	Yes	94 (75.2)	116 (59.8)	
Do you think the Presidency of Religious Affairs has a fatwa on organ donation?	No	2 (1.6)	5 (2.6)	0.101
	No idea	68 (54.4)	127 (65.1)	
	Yes	55 (44.0)	63 (32.3)	
Do you think it is appropriate for a non-Muslim's organs to be used for a Muslim?	No	5 (3.97)	4 (2.1)	0.216
	No idea	17 (13.5)	39 (20.0)	
	Yes	104 (82.5)	152 (77.95)	
Do you think it is appropriate for a Muslim's organs to be used for a non-Muslim?	No	5 (4.0)	5 (2.6)	0.714
	No idea	20 (16.0)	35 (18.0)	
	Yes	100 (80.0)	155 (79.5)	
Do you think brain death can be reversible?	No	68 (54.0)	116 (59.8)	0.563
	No idea	29 (23.0)	41 (21.1)	
	Yes	29 (23.0)	37 (19.1)	

Table 10 demonstrates the distribution of responses related to organ donation knowledge and attitudes according to the postgraduate education program. Only the response to the question, “Do you think the Presidency of Religious Affairs has a fatwa on organ donation?” revealed a statistically significant difference (*p* = 0.010) between educational programs. Specifically, 43.3% of doctoral students answered “yes,” while 68.2% of master's students indicated “no idea.”

**Table 10:** Comparison of knowledge and attitudes of postgraduate students regarding organ donation according to education programs.

**Items**	**Response**	**Master's degree [n (%)]**	**Doctorate degree [n (%)]**	***p***
Do religious beliefs have a place in your life?	No	10 (7.5)	13 (7.2)	0.925
	Yes	124 (92.5)	168 (92.8)	
Do your religious beliefs influence your important decisions?	No	14 (10.4)	14 (7.8)	0.107
	Partially	46 (34.1)	45 (25.0)	
	Yes	75 (55.6)	121 (67.2)	
Do you think organ donation is appropriate for Islamic belief?	No	12 (8.9)	11 (6.2)	0.634
	No idea	36 (26.7)	47 (26.3)	
	Yes	87 (64.4)	121 (67.6)	
Do you think the Presidency of Religious Affairs has a fatwa on organ donation?	No	5 (3.7)	2 (1.1)	0.010
	No idea	92 (68.2)	100 (55.6)	
	Yes	38 (28.2)	78 (43.3)	
Do you think it is appropriate for a non-Muslim's organs to be used for a Muslim?	No	4 (3.0)	5 (2.8)	0.982
	No idea	24 (17.8)	31 (17.1)	
	Yes	107 (79.3)	145 (80.1)	
Do you think it is appropriate for a Muslim's organs to be used for a non-Muslim?	No	5 (3.7)	5 (2.8)	0.898
	No idea	23 (17.0)	31 (17.2)	
	Yes	107 (79.3)	144 (80.0)	
Do you think brain death can be reversible?	No	80 (59.7)	101 (55.8)	0.216
	No idea	32 (23.9)	36 (19.9)	
	Yes	22 (16.4)	44 (24.3)	

Table 11 outlines the distribution of postgraduate students' responses to questions evaluating their knowledge and attitudes toward organ donation, with a specific focus on their willingness to donate organs. The analysis revealed a statistically significant difference between the groups' responses to the following questions: (i) “Do you think organ donation is appropriate for Islamic belief?” (*p* < 0.001), (ii) “Do you think it is appropriate for a Muslim's organs to be used for a non-Muslim?” (*p* = 0.004), (iii) “Do you think brain death can be reversible?” (*p* < 0.001).

**Table 11:** Comparison of knowledge and attitudes of postgraduate students regarding organ donation by response to question of “Have you donated your organs.”

**Items**	**Response**	**Have you donated your organs? [n (%)]**			***p***
		**Undecided**	**I don't think so**	**Yes**	
Do religious beliefs have a place in your life?	No	14 (6.2)	4 (10.0)	6 (11.1)	0.387
	Yes	211 (93.8)	36 (90.0)	48 (88.9)	
Do your religious beliefs influence your important decisions?	No	15 (6.7)	6 (14.6)	8 (14.8)	0.066
	Partially	69 (30.8)	6 (14.6)	16 (29.6)	
	Yes	140 (62.5)	29 (70.7)	30 (55.6)	
Do you think organ donation is appropriate for Islamic belief?	No	14 (6.3)	8 (19.5)	1 (1.9)	< 0.001
	No idea	57 (25.5)	19 (46.3)	10 (18.9)	
	Yes	153 (68.3)	14 (34.2)	42 (79.3)	
Do you think the Presidency of Religious Affairs has a fatwa on organ donation?	No	6 (2.7)	1 (2.4)	0 (0.0)	0.185
	No idea	142 (63.1)	26 (63.4)	26 (49.1)	
	Yes	77 (34.2)	14 (34.15)	27 (50.9)	
Do you think it is appropriate for a non-Muslim's organs to be used for a Muslim?	No	7 (3.1)	2 (4.9)	0 (0.0)	0.107
	No idea	37 (16.4)	12 (29.3)	7 (13.0)	
	Yes	181 (80.4)	27 (65.9)	47 (87.0)	
Do you think it is appropriate for a Muslim's organs to be used for a non-Muslim?	No	5 (2.2)	4 (9.8)	1 (1.9)	0.004
	No idea	36 (16.0)	13 (31.71)	6 (11.3)	
	Yes	184 (81.8)	24 (58.5)	46 (86.8)	
Do you think brain death can be reversible?	No	135 (60.3)	10 (24.4)	39 (72.2)	< 0.001
	No idea	47 (21.0)	14 (34.15)	8 (14.8)	
	Yes	42 (18.8)	17 (41.5)	7 (13.0)	

Table 12 summarizes the Cronbach's alpha coefficients, representing the levels of internal consistency and reliability obtained from both the total and subdimensions of the ODAS scale. The Cronbach's alpha value for the HMC subdimension is 0.945, for the FMN subdimension is 0.863, for the FBM subdimension is 0.817, and for the total dimension is 0.841. These values indicate a high level of internal consistency and reliability within the ODAS scale.

**Table 12:** Internal reliability analysis of total and subdimension questions of ODAS scale.

**Variables**	**Cronbach's alpha**
HMC score	0.945
FMN score	0.863
FBM score	0.817
Total score	0.841

HMC, Humanity and moral convinction; FMN, Fears of medical neglect; FBM, Fears of bodily mutilation.

Table 13 depicts the correlation between the ODAS total score and the subdimension scores (HMC, FMN, and FBM), along with the statistical significance levels of these correlations. The correlation coefficient (r) between the ODAS total score and HMC score is 0.503 (*p* < 0.001). Likewise, the correlation between ODAS total score and FMN score is 0.605 (*p* < 0.001), and between ODAS total score and FBM score is 0.525 (*p* < 0.001). Notably, the correlation between the HMC score and FMN score is negative (r = −0.149) and statistically significant (*p* = 0.008), as is the correlation between FBM and HMC score (r = −0.295, *p* < 0.001). In contrast, the correlation between the FMN score and the FBM score is positive and highly significant (*r* = 0.592, *p* < 0.001).

**Table 13:** Correlation analysis of total and subdimension scores of ODAS scale.

**Variables**	**Correlation coefficient (r) and p-value**	**Total score**	**HMC score**	**FMN score**	**FBM score**
Total score	r	1.000	0.503	0.605	0.525
	p	.	< 0.001	< 0.001	< 0.001
HMC score	r	0.503	1.000	−0.149	−0.295
	p	< 0.001	.	0.008	< 0.001
FMN score	r	0.605	−0.149	1.000	0.592
	p	< 0.001	0.008	.	< 0.001
FBM score	r	0.525	−0.0295	0.592	1.000
	p	< 0.001	< 0.001	< 0.001	.

HMC, Humanity and moral convinction; FMN, Fears of medical neglect; FBM, Fears of bodily mutilation.

## 4 Discussion

The shortage of organ donors constitutes a significant problem for healthcare systems worldwide, resulting in longer waiting times for patients requiring organ transplants and increased mortality rates while on the waiting list, particularly for patients requiring vital organ transplants such as liver, heart, and lung (27). Organ donation is a vital aspect of modern healthcare and significantly impacts the lives of patients with end-stage organ failure. However, organ donation rates remain critically low in many regions, including Turkey, necessitating a comprehensive investigation into the factors influencing public attitudes and behaviors toward organ donation—a question that underlies this present study. Understanding the factors that influence knowledge, attitudes, and behaviors toward organ donation is crucial to developing effective strategies to increase donor rates and alleviate the organ shortage crisis. One such influential factor is religious beliefs, which play a central role in shaping individuals' perspectives on life, death, and altruistic acts such as organ donation (28). There are studies in the literature that investigate the relationship between religion and organ donation attitudes across various faith groups, geographic regions, and demographic groups (29–31).

While some studies have noted a positive association between religiosity and opposition to organ donation due to beliefs about bodily integrity and concerns about the afterlife, others have found that religious doctrines may also support altruistic acts such as organ donation, thus promoting positive attitudes within religious communities (20). However, the majority of existing studies on organ donation attitudes prioritize the general population, thereby paying limited attention to the perspectives of specific demographic groups like postgraduate students. The findings of this study, which was conducted to evaluate the relationship between organ donation attitudes and religious beliefs of postgraduate students who have a certain level of education and who will play an important role in shaping the perspectives of society and educational institutions, were discussed in line with the relevant literature.

Examining the study's findings reveals that young adult women (61.1%) constitute the majority of students, with 89.7% studying in programs directly related to health sciences, and more than half (57.4%) are doctoral students. Examining the studies conducted on undergraduate and graduate students reveals that the female gender, age, and marital status vary from study to study, and as expected, these rates will rise in tandem with the duration of education (1, 3, 32, 33). Examining the literature reveals a prevalent use of “postgraduate” in place of “graduate.” Therefore, the literature lacks sufficient data comparing master's and doctoral students in this context, or in general, regarding the relationship between organ donation awareness and beliefs among postgraduate students.

Examining the students' views on organ donation revealed that while 96.5% believed it was necessary, only 16.9% actually donated their organs. The discrepancy between high positive attitude scores and low actual organ donation rates observed in this study may be attributed to cultural and religious influences that shape individual perceptions. Although postgraduate students express positive views toward organ donation, practical willingness remains limited due to religious considerations and concerns regarding bodily integrity. Similar results were found in the study conducted by Akbulut et al. (13–15) where a significant number of postgraduate students agreed on the necessity of organ donation, yet the actual donation rates remained very low, highlighting a paradox that warrants further investigation.

Bapat et al. (33) conducted a study on medical students and found that while 89% of the participants were willing to donate their organs, only a small number actually did so. The authors suggested that the reasons for this situation included fears, concerns about the accuracy of brain death diagnosis, and religious beliefs. We believe that religious beliefs are effective in making some important decisions on this issue. In fact, in the study we present here, 62.2% of the postgraduate students stated that their religious beliefs were effective when making decisions in daily life, while 28.7% stated that their beliefs partially affected their decisions. Therefore, we can conclude that even in Islamic societies with high levels of education, beliefs play a significant role in decision-making, including the issue of organ donation. Chu et al. (34) conducted a study on medical students in Hong Kong, revealing that almost all of them had a positive attitude toward organ donation. However, only 28.1% of the students were organ donors, citing their belief in maintaining body integrity after death as one of their reasons.

Regarding organ donation, it's crucial to understand that both medically and legally, brain death is defined as the irreversible cessation of all brain activity, including the brain stem, which is crucial for maintaining life (35). In the present study, 57.5% of postgraduate students expressed their belief that a person who has experienced brain death cannot come back to life. However, it is important to note that 20.6% of the postgraduate students, who had a high level of education, responded affirmatively to this statement. We believe that the majority of postgraduate students believed in the irreversibility of brain death due to their background in health sciences and familiarity with health-related concepts. Similar to the present study's results, a study on the general population in India found that most participants answered negatively when asked about the possibility of a person's return to life (36). In a study by Akbulut et al. (15) covering the general Turkish population, 66% of the participants stated that brain death was irreversible, and the level of education of most of these participants was lower than the current study, indicating that there is not always a correlation between the level of education and the level of awareness and knowledge about organ donation.

The data from the scales evaluating the postgraduate students' attitudes toward organ donation reveals that most participants view organ donation as positive, albeit with some concerns. While a significant portion of the participants believe that an individual who accepts to donate their organs after death adds extra meaning to their life, some participants view organ donation as a moral behavior. Similarly, a Turkish study found that while a large portion of participants do not donate organs, they are willing to do so; most individuals who donate or wish to donate organs do so to save a life, serve humanity, or assist someone else (37). However, some participants stated that thinking about organ donation brings unpleasant thoughts about their own death and that they are concerned that organ donation may lead to a decrease in medical treatment. Dick et al. (38) noted in their study that the organ donation process may involve experiences of grief, ethical dilemmas, vicarious trauma, or compassion fatigue, potentially including unpleasant thoughts about death, for both the potential donor's family and members of the multidisciplinary team.

The present study evaluated postgraduate students' knowledge, attitudes, and behaviors regarding organ donation using the total, negative attitude subdimensions (FMN and FBM), and positive attitude subdimension (HMC) of the ODAS scale. In this study, it was determined that there was a difference between the master's and doctoral students in terms of HMC score, and this difference was higher in favor of doctoral students (p < 0.05). Majeed et al. (39) conducted a study on nursing students and found a statistically significant difference between the students' class and the positive attitude subdimension of the ODAS scale. Yakar et al. (40) found a significant difference in the HMC subdimension based on the level of education. Sengül and Sahin (41) conducted a study on medical students, revealing a significant difference in the positive attitude subdimension of the ODAS scale based on the possession of an organ donation card. Tas et al. (28) conducted a study that found no statistically significant relationship between marital status, educational level, approval of organ donation, and HMC score. However, they discovered a statistically significant correlation between factors such as gender, family approval of organ transplants, approval of organ donation based on Islamic beliefs, contemplation of organ donation, approval of organ transplantation in case of need, and HMC score.

We evaluated in detail the effects of postgraduate students' religious beliefs, gender, and education levels on organ donation attitudes. We determined no statistically significant difference between gender and education program categories in terms of organ donation attitudes (*p* > 0.05). Although there are studies investigating the relationship between gender, religious beliefs, and attitudes toward organ donation, the specific effect of gender may vary according to studies (23, 42). There are few studies examining the interaction between gender, religious beliefs, and attitudes toward organ donation, especially among students (43). The study by Tas et al. (28) reveals a significantly higher positive attitude score in women.

Measuring the reliability and internal consistency of the responses to the survey questions is an indicator of whether the participants read them carefully. This study found the Cronbach's alpha coefficients for the ODAS total, HMC, FMN, and FBM subdimensions to be 0.841, 0.945, 0.863, and 0.817, respectively. This indicates a sufficient understanding of the survey. Tas et al. (28) calculated the Cronbach's alpha for the positive and negative subdimensions to be 0.910 and 0.890, respectively, and these results are in line with the present study.

### 4.1 Limitations

This study has several limitations that should be acknowledged. (i) Firstly, the limited diversity of the sample, predominantly consisting of postgraduate students from a single academic institution, may restrict the generalizability of the findings. Incorporating data from multiple centers and diverse regions would enhance the robustness of the findings and allow for the exploration of potential regional and cultural differences in organ donation attitudes and religious beliefs. Additionally, comparing students from health sciences institutes with those from non-health sciences institutes would have provided a more comprehensive perspective on the factors influencing organ donation attitudes. However, due to difficulties in accessing students from other institutes, such comparisons could not be made. (ii) Another limitation of this study is its cross-sectional design, which limits the ability to establish causal relationships between organ donation attitudes and the influencing factors. The reliance on self-reported data may also introduce response bias, as participants might have answered in a socially desirable manner. (iii) Lastly, although the *post-hoc* power analysis indicated a high power for the study, an a priori power analysis and sample size calculation would have been preferable to ensure a more robust study design. Despite these limitations, the study provides valuable insights into the relationship between organ donation attitudes and religious beliefs among postgraduate students, highlighting the need for further research in more diverse and multi-centered contexts.

## 5 Conclusion

The relationship between organ donation attitudes and religious beliefs is complex and multifaceted. Considering the significant influence of religious beliefs on attitudes toward organ donation, it is essential to integrate religious perspectives when organizing awareness and education campaigns. In particular, collaborating with religious leaders can positively shape public perceptions and increase donation rates, as their views and declarations hold considerable sway in guiding societal attitudes. Therefore, it is crucial to design awareness programs that respect various religious beliefs and cultural values while promoting organ donation.

## Data Availability

The raw data supporting the conclusions of this article will be made available by the authors, without undue reservation.
